# Evaluating the Dimensionality of the Sociocultural Adaptation Scale in a Sample of International Students Sojourning in Los Angeles: Which Difference between Eastern and Western Culture?

**DOI:** 10.3390/ejihpe12050035

**Published:** 2022-05-18

**Authors:** Giusy Danila Valenti, Paola Magnano, Palmira Faraci

**Affiliations:** 1 Department of Psychology, Educational Science and Human Movement, University of Palermo, 90128 Palermo, Italy; 2 Faculty of Human and Social Sciences, University of Enna “Kore”, 94100 Enna, Italy; paola.magnano@unikore.it (P.M.); palmira.faraci@unikore.it (P.F.)

**Keywords:** sociocultural adaptation, sociocultural competence, factorial structure, measurement invariance

## Abstract

The Sociocultural Adaptation Scale (SCAS) measures the degree of sociocultural competence in new cultural settings, and, despite its popularity, research aiming at evaluating its dimensionality is lacking and has incongruent results. Moreover, the dimensionality of the scale has been mainly tested on different samples adjusted to Eastern culture. We administered the SCAS to 266 international students sojourning in Los Angeles to test which underlying dimensionality emerges if the measure is used to assess sociocultural adaptation to Western culture, also verifying its measurement invariance across sex. Findings from EFA showed a three-factor solution: *Diversity Approach*, *Social Functioning*, and *Distance and Life Changes*, and the CFA indicated a plausible goodness-of-fit to the empirical data. The examination of MGCFA suggested that the questionnaire showed an invariant structure across sex. Our results suggest that the dimensionality of the SCAS may differ according to the sojourners’ country of settlement, emphasizing Western–Eastern differences.

## 1. Introduction

In cross-cultural psychology research, there seems to be a wide agreement about the dichotomization of adaptation into two different and interrelated core domains: psychological and sociocultural [1]. The former refers to feelings such as satisfaction or well-being, and it is affected by personality variables, coping styles, and social support [2,3,4], the latter, defined in terms of behavioral competence, concerns the ability to fit in and to acquire culturally appropriate skills of the host environment, and it is mainly predicted by cultural knowledge, cultural distance, degree of contact, and positive intergroup attitudes [2,5].

When considering their conceptual and empirical distinction, psychological and sociocultural adaptation is measured by different assessment scales. The psychological domain is primarily evaluated using standard psychological assessment measures, such as the well-known Satisfaction with Life Scale [6], the Self-Esteem Scale [7], the General Health Questionnaire [8], or the Well-Being Profile (WB-Pro), recently developed by Marsh and colleagues [9]. Instead, the sociocultural domain is predominantly assessed by the Sociocultural Adaptation Scale (SCAS; [5]), inspired by Furnham and Bochner’s [10] Social Situations Questionnaire (SSQ).

The SCAS is focused on assessing the competencies that are required to manage everyday social situations in new cultural contexts. This implies that sociocultural adjustment is meant as the acquisition of the behavioral skills to operate effectively in a new cultural environment, and it is measured in terms of the degree of self-reported difficulty experienced in interpersonal situations and with the attainment of day-to-day tasks [11]. The measure is flexible, versatile, and easily changeable according to the characteristics of the sojourning sample and the appropriateness of the specific cultural settings in which the study takes place [5]. Indeed, even if the original scale consists of 41 items, versions of different lengths are very common, and the most popular contain 20–23 items [4].

Driven by the culture-learning framework, the SCAS can be considered the first scale addressing the diverse and inconsistent ways in which sojourners’ adjustment had been previously conceptualized, assessed, and interpreted [11], and since its development, it has been largely used across different disciplines, including acculturation, cross-cultural transition, communication and language acquisition, education, international business and management, and organizational psychology [12,13,14,15,16,17].

Although a revised version of the SCAS has been recently validated (SCAS-R; [11]), the original version of the scale is still used [18,19,20], showing that the SCAS has not been fully replaced by its revised form and highlighting that its application continues to be extensive and widely common also in current studies.

In contrast with the large number of studies in which the SCAS has been used, research about its dimensionality is lacking, with incongruent and discrepant results. As an example, the factorial structure of the 29-item version of the scale, examined with the application of exploratory factor analysis, reported two underlying factors: The first factor was composed of cognitive and interpersonal relationships items (e.g., “Understanding the local value system”; “Making friends”), whereas the second factor described the amount of difficulty experienced in impersonal tasks and activities (e.g., “Dealing with bureaucracy”; “Dealing with someone who is unpleasant”). The two factors were labeled *Cultural Empathy and Relatedness* and *Impersonal Endeavours and Perils*, respectively [5]. Afterward, Chen and Choi [21] discovered that for Chinese immigrants in Singapore, three factors underlie sociocultural adaptation: *Social*, *Physical*, and *Cultural Adaptation*. Further, findings from Tanaka’s [22] research reported a three-factorial structure of a 25-item SCAS version, applied to evaluate sociocultural adjustment among international students in Japan: Factor 1 (*Academic Adaptation*) composed of items regarding issues at university; Factor 2 (*Survival*) describing difficulties in organizing daily life; Factor 3 (*Interpersonal Adaptation*) dealing with difficulties related to social interactions. Following, Renner, Salem, and Menschik-Bendele [23] conducted a study aimed at testing the factorial structure of a modified version of the SCAS (30 items) used for assessing German students’ adaptation in Austria. Three dimensions emerged from their study: Factor 1 (*Mentality*) dealing with understanding Austrian culture, mentality, and values; Factor 2 (*Communication*) concerning communication issues; Factor 3 (*Studies and University*) describing academic requirements at the university. However, in this study, five items that addressed specific problems German students might encounter in Austria were added (e.g., “Dealing with the Austrian conception of achievement and success” and “Understanding Austrians’ need for harmony”) to make the questionnaire more tailored to the target population.

The results from these factorial analyses cannot be easily compared to each other both for the different sojourning samples to which the scale was administered and for the diverse content and number of items taken into account. The only shared feature of the aforementioned studies consists in relying only on exploratory factor analysis examinations. Additionally, the SCAS dimensionality has been mainly tested on different samples adjusted to Eastern culture. Thus, we aimed to evaluate the factorial structure of the SCAS in order to examine whether the underlying dimensionality of the measure differs if it is used to assess sociocultural adaptation to Western culture.

As mentioned above, and as described in these empirical studies, the SCAS shows a high degree of versatility and flexibility. This aspect can be considered as a double-edged sword: in fact, on the one hand, choosing which items fit best to the specific cultural context and sojourning sample could be advantageous because flexibility allows for creating a suitable and appropriate measure for any kind of situation; on the other hand, this could represent a weakness, provided that the extreme ease in modifying the length and the content of the scale makes the SCAS a poorly stable and scarcely solid measure, and cross-sectional comparisons become harder and more complex. Actually, Ward and Kennedy [5] reported good reliability and validity in 16 cross-sectional studies, but they were based only on the 10 shared items. That is, the questionnaires used in these 16 studies differed from each other for the content and/or the number of items, and only 10 items were consistent across the different versions of the scale. Ward and Kennedy [5] conducted their study on the 10 items the questionnaires had in common.

In other words, the current literature highlights a lack of coherence in sociocultural adjustment evaluation through the SCAS, and, especially, there seems to emerge a paucity of studies specifically aimed at deeply investigating its psychometric properties by using robust and sophisticated analytic techniques. Furthermore, these studies are based on exploratory study explorations. This work aims at filling this gap by extending the knowledge about sociocultural assessment in cross-cultural research and offering a contribution with the application of more powerful factorial structure investigations.

Thus, the main goal of the present study is to test the dimensionality of the SCAS by performing both exploratory and confirmatory factor analyses. To this end, a 20-item version of the scale was administered to 266 international students sojourning in Los Angeles, California. The choice of the SCAS version to be analyzed was guided by a relevant and recent international project—the Mutual Intercultural Relations In Plural Societies, MIRIPS (http://www.victoria.ac.nz/cacr/research/mirips, accessed on 14 December 2018)—focused on investigating the psychological aspects related to intercultural relations in multicultural contexts. The MIRIPS involves several countries, including Russia, Italy, Portugal, Finland, and Canada [24,25,26,27,28], and it shares a common research framework and the same research assessment tool.

None of the 20 items constituting the SCAS are context-dependent, and they can be easily used to assess the degree of sociocultural competence regardless of both the specific situation and sample examined. We argue that a robust and psychometrically sound scale should contain a well-defined number of items whose content should not be strongly tailored to any particular cultural context and sojourning population. This represents a key aspect for cross-cultural and cross-samples comparisons.

In this regard, we decided on purpose not to include items such as “Coping with academic work”, “Understanding what is required at university” or “Expressing your ideas in class” because we believe that, although they are suitable for the specific population investigated, these items may reflect the construct of academic adjustment [29] rather than the sociocultural adaptation. From our perspective, academic adjustment is a too broad concept to be assessed as a domain of sociocultural adaptation. Academic adjustment is rather a distinct concept from sociocultural adjustment, with specific predictors and outcomes. In line with this claim, while the sociocultural adjustment is predominantly affected by situational variables, such as cultural distance or the degree of contact between cultures [2], academic adjustment is mainly affected by personality variables, coping styles, self-esteem, or self-efficacy [30].

## 2. Materials and Methods

### 2.1. Participants and Procedure

The total group of participants consisted of 266 college students, 131 males (49.2%) and 135 females (50.8%), with a mean age of 24.23 (*SD* = 4.59), enrolled at UCLA (University of California, Los Angeles) as international students (*n* = 213), and at two English Languages Schools: Columbia West College (*n* = 23) and Kaplan International Languages College (*n* = 30). Data were collected individually or in small groups. Data at UCLA were gathered individually, through informal contacts with the international students met all around the campus, whereas at the two English Language Schools, the first author was introduced by the English teacher in the class, and the questionnaire was administered to small groups composed of 8–10 international students. Data collection was performed from January to March 2019.

### 2.2. Instrument

The Sociocultural Adaptation Scale (SCAS) consists of 20 items assessing the difficulties experienced by international students in the host society. It focuses on the needed skills to cope with everyday social situations encountered in a new culture (e.g., making friends, using public transportations, bureaucracy).

Participants were asked to indicate the extent to which each statement was true for them on a 5-point Likert scale (from 1 “No difficulty” to 5 “Extreme difficulty”). The final score was reversed, with higher scores indicating lesser problems and higher levels of sociocultural competence.

### 2.3. Data Analysis

Prior performing the data analyses to determine the factorial structure of the SCAS, several steps were taken for initial screening of the items’ distribution. The distributional properties of each item were examined by inspecting both skewness and kurtosis. The normality of the data was checked through Kolmogorov–Smirnov and Shapiro–Wilk tests.

The factorability of the data was examined by calculating the Kaiser–Meyer–Olkin (KMO) Measure of Sampling Adequacy. The KMO is an indicator of the amount of shared variance in the item pool; it can range from 0 to 1, and values greater than 0.80 should be considered good.

Principal Axis Factoring Exploratory Analysis (PAF-EFA) with Promax rotation was applied. In order to fix the number of factors, Kaiser’s criterion and Cattel’s scree test were checked. A Random Parallel Analysis, using Bryan O’ Connors [31] syntax, was also performed. The reliability of the scale, in terms of internal consistency, was computed by the computation of coefficient alpha.

Confirmatory factor analysis was conducted in order to determine the appropriateness of the proposed model using JASP 0.12.2 [32]. Several goodness-of-fit indexes were used to verify whether the fit was adequate to support the model: the ratio of the chi-square to degrees of freedom (χ^2^/df), the Non-Normed Fit Index (NNFI), the Comparative Fit Index (CFI), the Standardized Root Mean Square Residual (SRMR), and the Root Mean Square Error of Approximation (RMSEA). Hu and Bentler [33] highly recommend considering multiple measures in order to examine different aspects of fit.

A non-significant chi-square, Comparative Fit Index (CFI), and Non-Normed Fit Index (NNFI) above 0.90, and Root Mean Square Error Approximation (RMSEA) and Standardized Root Mean Square Residual (SRMR) below 0.05 have all been claimed as useful indicators of good fit [34]. Jöreskog and Sörbom [35] also stated that a chi-square statistic alone should not be used to support or reject a proposed model, also suggesting examining the chi-square/degrees of freedom (df) ratio.

Multigroup Confirmatory Factor Analyses (MCFAs) were run in JASP to evaluate whether the factor structure was invariant across sex. This method employs successive analyses where constraints to the models are added progressively.

Configural invariance, metric invariance, scalar invariance, and strict invariance across the groups were examined.

- *Configural invariance.* The first step in the process of testing invariance is to assess whether the same factor structure of the scale is supported in the groups (e.g., whether the same number of factors is relevant and whether the same items are salient to each factor). The configural invariance (Model A) is an unconstrained model in which parameters to be estimated are allowed to vary freely. It provides the basis for comparisons with all subsequent models in the invariance hierarchy.
- *Metric invariance*. Metric invariance (Model B) is obtained by adding constraints on the factor loadings to the base model. It tests the extent to which the relationships between the factors and the items are equivalent across groups. The χ^2^ difference test was then calculated to evaluate whether there was a significant difference between the constrained Model B and the unconstrained Model A.

If a non-significant difference between the two models is reported, it means that factor loadings are consistent across the groups [36]. However, because the χ^2^ difference test is not sufficient alone for evaluating overall model fit [37], the invariance hypothesis was further investigated by considering the discrepancies in the CFI between Model A and Model B. Metric invariance can be retained when the change in CFI is less than 0.01 [38].

C. *Scalar invariance*. If metric invariance is satisfied, scalar invariance can be tested by constraining the intercepts of items to be the same across groups (Model C). The χ^2^ difference test and the change in CFI were used to compare Model C to Model B: whenever non-significant differences between the two models are found, the intercepts are consistent across groups [36].
D. *Strict invariance*. Strict invariance (Model D) is examined by adding the residual equality constraint, indicating that the scale shows the same pattern of error variance between groups. As previously, in order to examine model invariance, we used ΔCFI and Δχ^2^ to compare Model D to Model C.

If measurement invariance is achieved, it can be assumed that the same dimensions are measured in the different groups and that the items function in the same way in both groups.

Although we are aware that a larger sample would have provided more reliable results, some authors [39] reported that in factor analyses, any N > 200 offers adequate statistical power for data analyses. In addition, for a multigroup CFA, a minimum of 100 participants in each group is recommended, as suggested previously [40,41]. From this perspective, our sample size can be considered suitable for our study.

## 3. Results

### 3.1. Item Distribution

In order to investigate the distributional properties of each item, skewness and kurtosis were firstly examined, and, in some cases, they exceeded |1|. Both Kolmogorov–Smirnov and Shapiro–Wilk tests of normality were also evaluated, and both of them were significant. These results suggested that item distribution was not normal (see Table 1). For this reason, in an effort to examine the underlying structure of the SCAS, estimation procedures not assuming the normal distribution were chosen, the principal axis factoring method was used for exploratory factor analysis, and the maximum likelihood robust estimation procedure was applied for confirmatory factor analysis. For a more detailed description of item distribution, please see Supplementary Materials.

### 3.2. Exploratory Factor Analysis

First of all, in order to evaluate if items were appropriate for factor analysis, both Barlett’s Test of Sphericity and Kaiser–Meyer–Olkin Measure of Sampling Adequacy were examined. Bartlett’s Test of Sphericity (χ^2^ = 2000.096; df = 190) was significant (*p* < 0.001), indicating that the correlation matrix was factorable based on a suitable level of variables interrelations, and the KMO was excellent at 0.90.

In order to fix the number of factors, we used parallel analysis, and the results indicated there were four factors to be extracted (see Table 2). Furthermore, the examination of eigenvalues and the scree plot suggested that four factors should be retained. Eigenvalues were evaluated using the eigenvalue rule, which suggests that factors with a value less than one should not be retained because they provide less information.

The results of the Principal Axis Factor extraction with Promax rotation showed a four-factor structure, with five items reporting a double loading (item 4 “Dealing with people in authority” loaded at 0.34 on F1 and at 0.54 on F3; item 6 “Dealing with bureaucracy” loaded at 0.50 on F3 and at 0.33 on F4; item 7 “Making yourself understood” loaded at 35 on F1 and at 0.31 on F2; item 9 “Understanding jokes and humour” loaded at 0.34 on F1 and at 0.54 on F4; item 17 “Talking about yourself with others” loaded at 0.44 on F1 and at 0.35 on F2). For this reason, they were deleted, resulting in a 15-item scale.

After removing these items, the analysis was rerun restricting the number of factors to three, and the resulting factor loadings were examined.

Factor 1 (6 items), with an eigenvalue of 5.73, accounted the 35.81% of the total variance, and it was composed of six items; Factor 2 (6 items) had an eigenvalue of 1.42 and it was responsible for the 9.43% of the total variance; F3 (3 items) had an eigenvalue of 1.23 and accounted for the 8.16% of the total variance. Items and factor loadings are shown in Table 3. Next, two additional items were removed from F2 due to a corrected item-total correlation below 0.40 (item 2, “Finding food that you enjoy”; item 10, “Obtaining accommodation”). Thus, the second factor consisted of four items. The resulting factors showed satisfactory reliability and good levels of corrected item-total correlations (Table 3). The final correlations among scale factors were acceptable. Specifically, the correlation between F1 and F2 was 0.63, the association between F1 and F3 was 0.55, and the Pearson’s *r* value in the relationship between F2 and F3 was 0.57.

After refining the subscales, a total of 13 items were retained. Upon the examination of the items’ loadings on each factor, labels for the three subscales were generated: The first factor was made of six items reflecting how individuals face diversity in the new sociocultural context, and it could be named Diversity Approach (e.g., “Understanding cultural differences”; “Communicating with people of a different ethnic group”); the second factor (items 1, 3, 5, 8) could be labeled Social Functioning because the items it was made of concern issues in following social rules or in handling social tasks (e.g., “Following rules and regulations”; “Using the transport system”); the third Factor (items 18, 19, 20), named Distance and Life Changes, involves items describing how individuals experience changes related to cross-cultural transition and how they feel living far away from home (e.g., “Dealing with the climate”; “Family relationships”).

### 3.3. Confirmatory Factor Analysis

A confirmatory factor analysis was performed to determine whether the three-factor model of the SCAS fitted the data. The latent variables have been allowed to correlate with each other. As indicated earlier, the assumption of multivariate normality was violated, and, consequently, the robust bethod of parameter estimation was used. In order to evaluate the model fit, several fit indices were computed.

**Figure 1:**
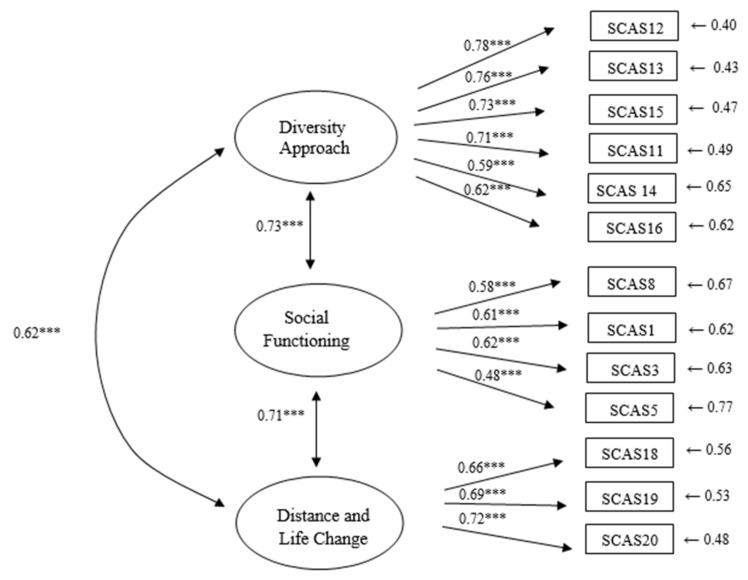
Standardized solution of the three-dimensional CFA model. Note. *** *p* < 0.001.

The χ^2^ was 137.70 on 62 degrees of freedom (*p* < 0.001), and the χ^2^/df ratio was 2.22. As shown in Table 4, this model provided a close to adequate fit for these data. The final CFI and NNFI were good at 0.93 and 0.92, respectively. The SRMR had a value of 0.05, indicating an acceptable fit, whereas the RMSEA was slightly above the good-fit range at 0.07 (90% confidence interval = 0.05–0.08). However, Steiger [42] considers RMSEA values less than 0.10 as an acceptable model fit. Table 4 shows the fit indices for the model. Figure 1 displays the standardized estimated parameters.

### 3.4. Test of Invariance

Invariance tests were performed for the 13-items 3-factors model. Invariance was assessed across sex in the following hierarchical ordering of nested models: configural invariance, metric invariance, scalar invariance, and strict invariance.

The first step consisted in evaluating the configural invariance (Model A), an unconstrained model in which parameters to be estimated were allowed to vary freely. Although a significant χ^2^ was obtained, all other fit indices were above the cut-off of acceptability. The configural invariance was then supported.

The equality constraints on factor loadings (Model B-metric invariance) yielded neither a significant increase of the χ^2^ nor change in CFI, suggesting that the relationships between the factors and the items were equivalent across groups.

After that, the scalar invariance (Model C) was tested by constraining the intercepts. As with the previous models, even if the χ^2^ value was significant, all other indices indicated an acceptable fit of the model. A non-significant χ^2^ difference and a discrepancy in CFI < 0.01 showed that the scalar invariance was satisfied.

Finally, the strict invariance was examined (Model D), in which the residual equality constraint was added to the previous model. Furthermore, the residual invariance model was accepted, not reporting neither significant differences in χ^2^ or discrepancies in CFI greater than 0.01.

In sum, these results provided evidence that the same dimensions were measured in the different groups and that the items functioned in the same way in both men and women. Fit indices for invariance tests across sex are displayed in Table 5.

We then performed independent samples *t*-test to investigate whether scores on the three factors differed between men and women. Results from our analyses showed that the levels of sociocultural adjustment were not statistically different in the two genders in none of the three emerged factors (Diversity Approach: *M*_men_ = 25.66, *SD* = 4.39, *M*_women_ = 25.66, *SD* = 3.97, *t*_(264)_ = 0.009, *p* = 0.992; Social Functioning: *M*_men_ = 17.21, *SD* = 2.72, *M*_women_ = 17.13, *SD* = 2.09, *t*_(264)_ = 0.295, *p* = 0.768; Distance and Life Changes: *M*_men_ = 13.07, *SD* = 2.26, *M*_women_ = 13.27, *SD* = 1.84, *t*_(264)_ = −0.783, *p* = 0.434). 

## 4. Discussion

In times of uncertainty and changes in the economic and social environments, adaptation to different sociocultural contexts is a core ability: students and workers, more than in the past, are requested to be flexible in adjusting to the changing places in which they build their own lives and careers because they are involved in more frequent transitions. From this point of view, they are required to improve several psychological adaptation abilities, such as psychological adjustment or career adaptability, and to increase the level of sociocultural adaptation. Moreover, the multi-ethnic and multicultural asset of the third-millennium societies—which are different manifestations of cultural diversity—implies new adaptation abilities for individuals and groups: the members of these ‘new societies’ are asked to interact actively with individuals coming from diverse cultural models and to contribute to the creation of social thinking, which is reactive to cultural uniformity [43].

This study investigated the factorial structure of the Sociocultural Adaptation Scale (SCAS), “a reliable, valid and extremely versatile instrument for the measurement of intercultural competence or behavioural adaptability” [5] (p. 673). The factorial structure of the 20-item version of the questionnaire was examined in a sample of international students in Los Angeles by performing both exploratory and confirmatory factor analyses.

Overall, our findings showed that the SCAS has good psychometric properties in terms of structural validity and internal reliability, providing further evidence about its suitability in assessing sociocultural adjustment in new and different cultural settlements.

Specifically, results from analyses revealed three underlying dimensions: The first factor was composed of items reflecting how individuals face diversity in the new sociocultural context, and it was named *Diversity Approach;* the second factor was labeled *Social Functioning*, and it comprises items that concern issues in following social rules or in handling social tasks; the third factor, *Distance and Life Changes*, involves items describing how individuals experience changes related to cross-cultural transition.

The confirmatory factor analysis showed a plausible goodness-of-fit of the proposed model to the empirical data. Furthermore, the examination of the measurement invariance indicated that the questionnaire showed an invariant structure across sex. This means that there is no differential functioning of the 13-item SCAS scale according to sex and, therefore, the test’s structure is equivalent in both men and women.

Our results do not confirm the same factorial structure as the original version of the scale. Indeed, in Ward and Kennedy’s seminal work [5], only two factors—*Cultural Empathy and Relatedness* and *Impersonal Endeavours and Perils*—emerged. The existence of such a different underlying dimension of the SCAS may be attributed to the diverse sociocultural context in which the scale has been administered. Sure enough, the original version of the SCAS was used to assess how well different samples adjusted to oriental sociocultural contexts (especially Singapore and New Zealand), whereas the current study was aimed at examining the levels of sociocultural adaptation to one of the main occidental multicultural countries, that is the United States.

Eastern and Western cultures differ from each other in a large number of features, including the political and economic systems, the main values, norms, and traditions.

Generally speaking, Eastern cultures are more collectivistic, interdependent, hierarchical, and dialectic, whereas Western cultures are more individualistic, independent, egalitarian, and less context-dependent. These East–West differences can be considered as the result of the differential use of social and individual learning, and they represent two diverse ways of adapting to the changes in the characteristics of the environment [44].

In line with this premise, it is reasonable that adjusting to Western countries—rather than to Eastern ones—requires a specific sociocultural competence. This may be a plausible explanation considering that a high percentage of our sample was composed of Asian students, whose cultural background extremely differed from one of the countries of settlement.

From this point of view, our findings offer intriguing theoretical implications, focusing on the importance of taking into account the sociocultural settings in which sociocultural adaptation has to be evaluated.

From a practical perspective, our research highlights that the SCAS is a measure with good psychometric properties, suggesting not to totally replace it with its revised form (SCAS-R). Furthermore, even if we are aware that the SCAS-R proposed by Wilson et al. [11] overcomes some of the shortcomings of the original version of the scale, the SCAS is able to capture some relevant aspects which cannot emerge through the application of the revised form. As an example, the SCAS-R does not include items assessing whether the sojourners experience difficulties in performing religious practices (“Worshipping”) in the host country, neglecting a significant facet to be investigated when adjusting to a new sociocultural environment. Indeed, especially for some cultures, religion plays a crucial role in personal and social life, and not endorsing the religious practices of the host country may negatively impact the degree of sociocultural adjustment to the new cultural setting.

### Limitations and Suggestions for Future Works

Some limitations of the study should be mentioned. The major issue is related to the estimation method applied for CFA. That is, although it can be justifiable if ordinal variables are treated as continuous by analyzing Pearson’s correlation in factor analysis [45], the MLR procedure might not guarantee unbiased estimates when the assumption of normality is violated. Further, the study employed a convenience sample of international students currently residing in the U.S.A. Such a sampling method does not assure adequate representativeness of the population, which in turn limits generalization beyond the study population. Additionally, the sample was highly weighted toward students from China (approximately 40%), and this uneven sampling did not allow us to perform separate exploratory factor analyses and multi-sample confirmatory factor analyses in order to verify structural invariance for the country of origin. Sure enough, investigating whether the factorial structure of the SCAS was invariant across the different sojourners’ homelands would have offered a deeper knowledge of the questionnaire, also providing further information about its level of structural validity. In order to disentangle culturally based external and internal factors, it will also be important to administer the scale to various groups of international students that share internal cultural factors but sojourning in different external situations. These comparisons will be hugely helpful for fully articulating the several ways in which culture might impact the sociocultural adaptation experience [46].

Additional research is needed and highly recommended aimed at further evaluating the underlying structure of the SCAS. We suggest that future investigations of the factorial dimensionality of the questionnaire should take into account the 20-item version, whose items can be easily used for any kind of sojourning sample. Future studies could compare different samples coming from unique countries, both from Eastern and Western cultural contexts. In addition, the outcomes from the current study need to be replicated and expanded, considering larger and diverse samples, in order to evaluate if the same dimensionality of the scale emerges. Finally, we suggest that future researchers, when testing the dimensionality of the SCAS, consider possible external factors that may affect the response to the questionnaire. For instance, the spread of the Covid-19 pandemic and the proliferation of several rules and regulations may influence the way people adapt to new and diverse sociocultural contexts. From this point of view, it is worthy to highlight that data collection was conducted prior to the diffusion of the virus, whereas works performed in the current period should pay attention to the situational circumstances affecting the country of settlement when interpreting results.

## 5. Conclusions

Our study provides a contribution to the examination of the dimensionality of the SCAS, also offering an insight into the assessment and interpretation of sociocultural adjustment when the host culture is a Western country. The results from our research showed that the SCAS is a good instrument to detect individual strengths and weaknesses in sociocultural adaptation, measured by its three dimensions, *Diversity Approach*, *Social Functioning*, and *Distance and Life Changes.* Although none of the SCAS items are strictly related to a specific sociocultural context and, consequently, they could be used for assessing sociocultural adaptation regardless of the sojourning sample, and the characteristics of the country of settlement, the underlying structure of the questionnaire is changeable, depending on we deal with sociocultural adjustment to Eastern or in Western culture. We humbly state that focusing on some of the general features that usually discriminate Eastern from Western cultures—such as the difference between collectivistic, interdependent, hierarchical, and dialectic society on the one hand and individualistic, independent, egalitarian, and less context-dependent society on the other hand—is a relevant aspect to be considered when assessing sociocultural adjustment. This topic, which is often neglected in the current literature, may be considered the key element for a better understanding of sociocultural adjustment conceptualization.

## Figures and Tables

**Table 1:** Item analysis for SCAS items.

Sample (*N* = 266)	*M*	*SD*	*S.E.*	Confidence Intervals 95%		S	K	K–S	S–W
				*LL*	*UL*				
Item 1	3.80	0.97	0.06	3.68	3.92	−0.49	−0.29	0.22 ***	0.86 ***
Item 2	4.19	1.07	0.07	4.06	4.33	−1.19	0.47	0.32 ***	0.75 ***
Item 3	4.51	0.79	0.05	4.32	4.61	−1.73	2.72	0.38 ***	0.66 ***
Item 4	4.30	0.88	0.07	4.20	4.40	−1.19	0.97	0.21 ***	0.76 ***
Item 5	4.14	1.05	0.05	4.01	4.27	−1.28	1.06	0.26 ***	0.77 ***
Item 6	3.81	1.07	0.06	3.70	3.94	−0.68	−0.31	0.22 ***	0.87 ***
Item 7	4.21	0.85	0.05	4.10	4.31	−0.97	−0.80	0.46 ***	0.80 ***
Item 8	4.71	0.64	0.04	4.64	4.79	−2.90	10.37	0.26 ***	0.50 ***
Item 9	3.88	1.09	0.07	3.74	4.01	−0.75	−0.26	0.23 ***	0.85 ***
Item 10	4.11	0.95	0.06	3.99	4.22	−0.82	−0.09	0.25 ***	0.82 ***
Item 11	4.00	0.95	0.06	3.89	4.11	−0.76	0.20	0.22 ***	0.84 ***
Item 12	4.26	0.84	0.05	4.15	4.35	−1.02	0.69	0.28 ***	0.79 ***
Item 13	4.22	0.86	0.05	4.12	4.32	−0.99	0.55	0.27 ***	0.80 ***
Item 14	4.34	0.94	0.06	4.22	4.45	−1.50	1.80	0.34 ***	0.72 ***
Item 15	4.42	0.88	0.05	4.32	4.53	−1.80	3.25	0.35 ***	0.68 ***
Item 16	4.35	0.82	0.05	4.25	4.45	−1.22	1.16	0.31 ***	0.75 ***
Item 17	4.26	0.96	0.06	4.14	4.38	−1.34	1.38	0.30 ***	0.75 ***
Item 18	4.59	0.73	0.04	4.50	4.67	−2.05	4.74	0.42 ***	0.61 ***
Item 19	4.36	0.91	0.05	4.26	4.46	−1.54	2.26	0.34 ***	0.71 ***
Item 20	4.21	0.90	0.06	4.10	4.32	−1.02	0.51	0.28 ***	0.79 ***

Note. *M* = Mean; *SD* = Standard Deviation; S = Skewness; K = Kurtosis; K–S = Kolmogorov–Smirnov test of normality; S–W = Shapiro–Wilk test of normality. Standard errors are computed with the bootstrap method (1000 bootstrap samples). *** *p* < 0.001.

**Table 2:** Raw Data Eigenvalues, Mean and Percentile Random Data Eigenvalues.

Root	Raw Data	Means	95% Percentil
1	6.56	0.60	0.70
2	1.00	0.50	0.57
3	0.76	0.42	0.49
4	0.48	0.36	0.42
5	0.32	0.30	0.35
6	0.23	0.25	0.29
7	0.20	0.19	0.24
8	0.10	0.15	0.19
9	0.06	0.10	0.14
10	0.03	0.06	0.09
11	−0.02	0.02	0.05
12	−0.03	−0.01	0.01
13	−0.04	−0.05	0.02
14	−0.10	−0.09	−0.06
15	−0.14	−0.13	−0.10
16	−0.15	−0.17	−0.14
17	−0.19	−0.20	−0.17
18	−0.20	−0.24	−0.21
19	−0.23	−0.29	−0.25
20	−0.27	−0.33	−0.30

**Table 3:** Factor loadings and indicators of reliability of the SCAS items.

	Factors		
	1	2	3
12. Communicating with people of a different ethnic group	**0.89** (0.71)	−0.21	0.07
13. Understanding ethnic or cultural differences	**0.83** (0.69)	−0.06	−0.05
15. Relating to members of the opposite sex	**0.71** (0.67)	0.02	0.01
11. Going to social gatherings	**0.67** (0.64)	0.19	−0.11
14. Worshipping	**0.51** (0.53)	−0.01	0.19
16. Finding your way around	**0.39** (0.54)	0.22	0.11
8. Going shopping	0.07	**0.52** (0.44)	0.14
1. Making friends	0.23	**0.50** (0.46)	−0.04
3. Following rules and regulations	0.09	**0.48** (0.48)	0.08
5. Using the transport system	−0.08	**0.47** (0.42)	0.14
18. Dealing with the climate	−0.12	−0.06	**0.74** (0.54)
19. Family relationships	0.03	−0.06	**0.73** (0.59)
20. The pace of life	0.02	−0.05	**0.49** (0.55)
% explained variance	35.81	9.43	8.16

Note. Factor loadings greater than 0.30 are displayed in bold; side loadings are shown in italics; item-total correlations are reported in parentheses, F1 = Diversity Approach; F2 = Social Functioning; F3 = Distance and Life Changes.

**Table 4:** Fit indices for the three-factors model.

χ^2^	df	*p*	χ^2^/df	NNFI	CFI	RMSEA	90% CI	SRMR
137.70	62	0.001	2.22	0.92	0.93	0.07	0.05–0.08	0.05

Note. NNFI = Non-Normed Fit Index; CFI = Comparative Fit Index; RMSEA = Root Mean Square Error of Approximation; SRMR = Standardized Root Mean Square Residual.

**Table 5:** Fit indices for invariant factor models.

Model	χ^2^	df	χ^2^/df	*p*	CFI	NNFI	SRMR	RMSEA	Δχ^2^ (Δχdf), *p*	ΔCFI
A. Configural	216.82	118	1.84	<0.001	0.92	0.89	0.06	0.08		
B. Metric	224.49	128	1.75	<0.001	0.92	0.90	0.07	0.07		
A–B Comparison									7.67 (10), *ns*	0.00
C. Scalar	237.01	138	1.72	<0.001	0.92	0.90	0.68	0.07		
B–C Comparison									12.52 (10), *ns*	0.00
D. Strict	256.79	153	1.68	<0.001	0.92	0.91	0.07	0.07		
C–D Comparison									19.78 (15), *ns*	0.00

Note. NNFI = Non-Normed Fit Index; CFI = Comparative Fit Index; RMSEA = Root Mean Square Error of Approximation; SRMR = Standardized Root Mean Square Residual; *ns =* not significant.

## Data Availability

The data used to support the findings of this study are available from the corresponding author upon request.

## Supplementary Materials

The following supporting information can be downloaded at: https://www.mdpi.com/article/10.3390/ejihpe12050035/s1, (A) Descriptives for each item; (B) Frequency distribution of each item; (C) Correlation matrix.
