# Supplementary material for: Evaluating the Dimensionality of the Sociocultural Adaptation Scale in a Sample of International Students Sojourning in Los Angeles: Which Difference between Eastern and Western Culture?
Source: Eur J Investig Health Psychol Educ. 2022 May 18;12(5):465–77. doi: 10.3390/ejihpe12050035 (PMC9142072; doi:10.3390/ejihpe12050035)
Supplement: Supplementary file 1 [file ejihpe-12-00035-s001.zip › ejihpe-1688887-supplementary.pdf]

Supplemental Materials

A) Descriptives for each item

Item	*M*	*SD*
Item 1	3.80	.97
Item 2	4.19	1.07
Item 3	4.51	.79
Item 4	4.30	.88
Item 5	4.14	1.05
Item 6	3.81	1.07
Item 7	4.21	.85
Item 8	4.71	.64
Item 9	3.88	1.09
Item 10	4.11	.95
Item 11	4.00	.95
Item 12	4.26	.84
Item 13	4.22	.86
Item 14	4.34	.94
Item 15	4.42	.88
Item 16	4.35	.82
Item 17	4.26	.96
Item 18	4.59	.73
Item 19	4.36	.91
Item 20	4.21	.90

B) Frequency distribution of each item

Item 1

	f	*f* %	*fc*
1	4	1.5	1.5
2	20	7.5	9.0
3	73	27.4	36.5
4	97	36.5	72.9
5	72	27.1	100
Tot.	266	100	

Item 2

	f	*f* %	*fc*
1	6	2.3	2.3
2	20	7.5	9.8
3	37	13.9	23.7
4	58	21.8	45.5
5	145	54.5	100
Tot.	266	100	

Item 3

	f	*f* %	*fc*
1	1	.4	.4
2	8	3.0	3.4
3	20	7.5	10.9
4	62	23.3	34.2
5	165	65.8	100
Tot.	266	100	

Item 4

	f	*f* %	*fc*
1	2	.8	.8
2	9	3.4	4.1
3	36	13.5	17.7
4	79	29.7	47.4
5	140	52.6	100
Tot.	266	100	

Item 5

	f	*f* %	*fc*
1	8	3.0	3.0
2	18	6.8	9.8
3	27	10.2	19.9
4	88	33.1	53.0
5	125	47.0	100
Tot.	266	100	

Item 6

	f	*f* %	*fc*
1	7	2.6	2.6
2	27	10.2	12.8
3	58	21.8	34.6
4	91	34.2	68.8
5	83	31.2	100
Tot.	266	100	

Item 7

	f	*f* %	*fc*
1	2	.8	.8
2	7	2.6	3.4
3	40	15.0	18.4
4	102	38.3	56.8
5	115	43.2	100
Tot.	266	100	

Item 8

	f	*f* %	*fc*
1	2	.8	.8
2	2	.8	1.5
3	9	3.4	4.9
4	44	16.5	21.5
5	209	78.6	100
Tot.	266	100	

Item 9

	f	*f* %	*fc*
1	7	2.6	2.6
2	28	10.5	13.2
3	49	18.4	31.6
4	89	33.5	65.0
5	93	35.0	100
Tot.	266	100	

Item 10

	f	*f* %	*fc*
1	2	.8	.8
2	15	5.6	6.4
3	51	19.2	25.6
4	83	31.2	56.8
5	115	43.2	100
Tot.	266	100	

Item 11

	f	*f* %	*fc*
1	4	1.5	1.5
2	13	4.9	6.4
3	57	21.4	27.8
4	98	36.8	64.7
5	94	35.3	100
Tot.	266	100	

Item 12

	f	*f* %	*fc*
1	1	.4	.4
2	9	3.4	3.8
3	34	12.8	16.5
4	99	37.2	53.8
5	123	46.2	100
Tot.	266	100	

Item 13

	f	*f* %	*fc*
1	1	.4	.4
2	11	4.1	4.5
3	35	13.2	17.7
4	100	37.6	55.3
5	119	44.7	100
Tot.	266	100	

Item 14

	f	*f* %	*fc*
1	4	1.5	1.5
2	12	4.5	6.0
3	27	10.2	16.2
4	70	26.3	42.5
5	153	57.5	100
Tot.	266	100	

Item 15

	f	*f* %	*fc*
1	4	1.5	1.5
2	9	3.4	4.9
3	18	6.8	11.7
4	74	27.8	39.5
5	161	60.5	100
Tot.	266	100	

Item 16

	f	*f* %	*fc*
1	1	.4	.4
2	8	3.0	3.4
3	29	10.9	14.3
4	87	32.7	47.0
5	141	53.0	100
Tot.	266	100	

Item 17

	f	*f* %	*fc*
1	5	1.9	1.9
2	11	4.1	6.0
3	34	12.8	18.8
4	76	28.6	47.4
5	140	52.6	100
Tot.	266	100	

Item 18

	f	*f* %	*fc*
1	2	.8	.8
2	2	.8	1.5
3	20	7.5	9.0
4	54	20.3	29.3
5	188	70.7	100
Tot.	266	100	

Item 19

	f	*f* %	*fc*
1	5	1.9	1.9
2	5	1.9	3.8
3	34	12.8	16.5
4	66	24.8	41.4
5	156	58.6	100
Tot.	266	100	

Item 20

	f	*f* %	*fc*
1	2	.8	.8
2	11	4.1	4.9
3	41	15.4	20.3
4	87	32.7	53.0
5	125	47.0	100
Tot.	266	100	

* Response rate from 1 “No difficulty” to 5 “Estreme difficulty"

C) Correlation matrix

	Item 1	Item 2	Item 3	Item 4	Item 5	Item 6	Item 7	Item 8	Item 9	Item 10	Item 11	Item 12	Item 13	Item 14	Item 15	Item 16	Item 17	Item 18	Item 19	Item 20
Item 1	-																			
Item 2	.29**	-																		
Item 3	.31**	.26**	-																	
Item 4	.31**	.27**	.56**	-																
Item 5	.25**	.22**	.34**	.34**	-															
Item 6	.23**	.22**	.36**	.40**	.43**	-														
Item 7	.40**	.10	.31**	.45**	.27**	.39**	-													
Item 8	.35**	.10	.43**	.31**	.28**	.26**	.26**	-												
Item 9	.34**	29**	.16*	.36*	.20**	.25**	.39**	.21**	-											
Item 10	.28**	.19**	.22**	.29**	.27**	.30**	.30**	.31**	.38**	-										
Item 11	.47**	.12	.31**	.43**	.21**	-22**	.43**	.25**	.42**	.37**	-									
Item 12	34**	.12	.30*	.42**	20**	.16*	.39**	.22**	.39**	.18**	.57**	-								
Item 13	.35**	.14*	.33**	.43**	.25**	.24**	.37**	.19**	.45**	.25**	.52**	.67**	-							
Item 14	.29**	.08	.28**	.35**	.25**	.18**	.29**	.23**	.37**	.18**	.42**	.47**	.47**	-						
Item 15	.44**	.06	.37**	.42**	.16**	.18**	.43**	.29**	.33**	.18**	.52**	.54**	.54**	.40**	-					
Item 16	.39**	.13*	.34**	.44**	.24**	.26**	.42**	.34**	.29**	.26**	.43**	.41**	.42**	.31**	.56**	-				
Item 17	.46**	.15*	.33**	.41**	.18**	.23**	.42**	.27**	.38**	.20**	.49**	.48**	.44**	.32**	.47**	.55**	-			
Item 18	.25**	.12	.26**	.27**	.26**	.22**	.25**	.33**	.18**	.08	.24**	.35**	.26**	.32**	.33**	.33**	.39**	-		
Item 19	.21**	.16*	.29**	.26**	.22**	.20**	.26**	.25**	.23**	.12	.29**	.32**	.29**	.28**	.26**	.29**	.35**	.50**	-	
Item 20	.44**	.17**	.28**	.28**	.33**	.25**	.37**	.29**	.29**	.23**	.37**	.31**	.32**	.29**	.32**	.40**	.52**	.44**	.50**	-

* $p < .05$; ** $p < .01$.
